# B Chromosomes and Cytogenetic Characteristics  of the Common Nase *Chondrostoma nasus* (Linnaeus, 1758)

**DOI:** 10.3390/genes11111317

**Published:** 2020-11-06

**Authors:** Alicja Boroń, Anna Grabowska, Aneta Spóz, Anna Przybył

**Affiliations:** Department of Zoology, Faculty of Biology and Biotechnology, University of Warmia and Mazury, M. Oczapowskiego St. 5, 10-718 Olsztyn, Poland; an.grabowska@nencki.edu.pl (A.G.); aneta.spoz@uwm.edu.pl (A.S.); anna.przybyl@uwm.edu.pl (A.P.)

**Keywords:** B chromosomes, C-banding, CMA_3_, Leuciscidae, FISH, rDNA sequences

## Abstract

Supernumerary B chromosomes (Bs) are very promising structures, among others, in that they are an additional genomic compartment for evolution. In this study, we tested the presence and frequency of B chromosomes and performed the first cytogenetic examination of the common nase (*Chondrostoma nasus*). We investigated the individuals from two populations in the Vistula River basin, in Poland, according to the chromosomal distribution of the C-bands and silver nucleolar organizer regions (Ag-NORs), using sequential staining with AgNO_3_ and chromomycin A_3_ (CMA_3_). Furthermore, we analyzed the chromosomal localization of two rDNA families (45S and 5S rDNA) using fluorescence in situ hybridization (FISH) with rDNA probes. *Chondrostoma nasus* individuals showed a standard (A) chromosome set consisting of 2*n* = 50: 12 metacentric, 32 submetacentric, and 6 acrocentric chromosomes (*NF* = 94). Fourteen out of the 20 analyzed individuals showed 1–2 mitotically unstable submetacentric B chromosomes of different sizes. Six of them, in 14.1% of the analyzed metaphase plates, had a single, medium-sized submetacentric B (Bsm) chromosome (2*n* = 51) with a heterochromatic block located in its pericentromeric region. The other seven individuals possessed a Bsm (2*n* = 51) in 19.4% of the analyzed metaphase plates, and a second Bsm chromosome (2*n* = 52), the smallest in the set, in 15.5% of metaphase plates, whereas one female was characterized by both Bsm chromosomes (2*n* = 52) in 14.3% of the analyzed metaphase plates. AgNORs, GC-rich DNA sites, and 28S rDNA hybridization sites were observed in the short arms of two submetacentric chromosome pairs of A set. The constitutive heterochromatin was visible as C bands in the centromeric regions of almost all *Chondrostoma nasus* chromosomes and in the pericentromeric region of several chromosome pairs. Two 5S rDNA hybridization sites in the pericentromeric position of the largest acrocentric chromosome pair were observed, whereas two other such sites in co-localization on a smaller pair of NOR chromosomes indicate a species-specific character. The results herein broaden our knowledge in the field of B chromosome distribution and molecular cytogenetics of *Chondrostoma nasus*: a freshwater species from the Leuciscidae family.

## 1. Introduction

B chromosomes (Bs), known as supernumerary or accessory chromosomes, are found in addition to standard A chromosomes, though they are not homologous to them. Because Bs are not essential parts of the genome, they vary in number among individuals, they are present in some, but not all, individuals, and are even present in certain cells of the same individual, but not in all [1,2]. Even if B chromosomes are not present in all individuals in the population, they may be present in most of the populations, such as is the case in the red-eye tetra, *Moenkhausia sanctaefilomenae* (Steindachner, 1907) [3]. Sometimes, their distribution seems to be sex dependent [4]. Supernumerary B chromosomes have been found in 2087 plants, 744 animals, and 14 fungi species [2,5,6]. Considering animals, the species possessing Bs are mainly insects (520 species), mammals (117 species), and ray-finned fish Actinopterygii (99 species) [5,7]. The presence of Bs may disturb the processes of mitosis and meiosis, and presumably because of this, only one or two such chromosomes are often recorded [1,6]. However, there are animal species with 5 or 10 Bs; the highest number being for the rodent *Apodemus peninsulae* (Muridae) with viz. 2*n* = 48 + 30B [2].

Among ray-finned fish, the occurrence of Bs is well documented in approximately 46 different taxa of neotropical fish from the order Characiformes, 28 species of Siluriformes, 19 different taxa of Perciformes, five Cypriniformes, and in a significantly lower number of other fish taxa [1,6]. The highest number of Bs (16) was observed in the cascarudo *Callichthys callichthys* (Linnaeus, 1758): a freshwater catfish from the family Callichthyidae distributed in South America [8]. Supernumerary chromosomes in fish vary greatly in size: from microchromosomes, such as in streaked prochilod *Prochilotus lineatus* (Valenciennes, 1837) [9], *M. sanctaefilomenae* [3], and Amazon molly *Poecilia formosa* (Girard, 1859) [10], through to medium-sized chromosomes, such as in *Rhamdia* species [11], to macrochromosomes detected among others in some populations of *Astyanax scabripinnis* (Jenyns, 1842) [12]. Macrochromosomes have been also found in two species from the family Leuciscidae [13]: the roach *Rutilus rutilus* (Linnaeus, 1758) [14,15] and bleak *Alburnus alburnus* (Linnaeus, 1758) [14,16,17]. In related cyprinid fish from the family Cyprinidae, such as the Prussian carp *Carassius gibelio* (Bloch, 1782) [18,19] and some hybrids of *Carassius* taxa [20], B microchromosomes have been detected. *A. alburnus* is distinguished by the fact that it possesses one of the largest B chromosomes ever found in vertebrates [17]. Furthermore, the B macrochromosomes of *A. scabripinnis* [12] and *R. rutilus* are similar in size to the A chromosomes [14].

B chromosomes can be derived intraspecifically from the A chromosome set of the host species, constituting a heterogonous mixture of genomic parasites as in *Astatotilapia latifasciata* (Regan, 1929) [21]. They can also be the result of evolutionary genome reduction as in Tetraodontidae [22] or from the sex chromosomes as in the characid fish *Characidium gomesi* [23]. Furthermore, they can result from interspecific hybridization, which provides foreign DNA from a closely related species [3,23] as in *A. alburnus* [14]. On the other hand, a portion of sex chromosomes in some cichlid species seems to be derived from B chromosomes [24].

The different staining techniques used in the initial period of B chromosome studies showed their heterochromatic nature [4,22]. The results of animals Bs investigated by chromosome mapping show that they are composed of different repetitive DNA sequences from the standard genome [21,23,25] and/or the B chromosome-specific DNA [14,17]. Moreover, recent findings from high-scale omics analyses indicated that studies of B chromosomes may contribute to the elucidation of many processes as they may affect the expression of genes located in A chromosomes [5]. Regardless of the techniques used, karyotyping is an essential step prior to genome sequencing to avoid problems in genome assembly and analytical biases created by the presence of high-copy-number sequences on the B chromosome [21].

The supernumerary chromosome has been described in the common nase *Chondrostoma nasus* (Linnaeus, 1758), which, like *R. rutilus* and *A. alburnus*, represents the leuciscid species from the subfamily Leuciscinae [13,26]. The common nase is widely distributed in Europe, but is invasive in some countries, and is locally threatened by damming, destruction of spawning sites, habitat loss, and pollution [27]. According to the limited data, *Chondrostoma nasus* is characterized by 2*n* = 50 chromosomes [28,29], similar as better cytogenetically known related species from the genus *Chondrostoma*, such as *C. lusitanicum* [30,31] and other Iberian chondrostomine taxa [32]. The exceptions are *Chondrostoma nasus* individuals from the Zeljeznica River in Bosnia and Herzegovina, possessing a karyotype of 2*n* = 51 chromosomes [26]. These data inspired the present study aiming to verify whether it was a random discovery, or whether the *Chondrostoma nasus* elsewhere in its range of distribution also have a single chromosome or perhaps more B chromosomes. We tested the presence and frequency of B chromosomes in the karyotype of *Chondrostoma nasus* from two populations in the Vistula River basin, in Poland. This study also presents the first cytogenetic characterization of *Chondrostoma nasus*, such as the chromosomal distribution of constitutive heterochromatin by C-banding, GC-rich DNA sites by CMA_3_ staining, nucleolar organizer regions (NORs) using the silver nitrate staining (AgNO_3_) and FISH mapping with 28S rDNA probe, and chromosomal localization of 5S rDNA. In this way, we provided new data about the structure of the A and B chromosome set of *Chondrostoma nasus* and enhanced the knowledge about the chromosomal and genomic diversity of the freshwater fish. The results add to the limited knowledge base of B chromosomes and their host species, of which *Chondrostoma nasus* is an example*,* with respect to global biodiversity.

## 2. Materials and Methods

Standard procedures for catching fish in aquaculture were employed during the study. Fish sampling and valid protocols for animal use in experiments were performed according to the European animal welfare recommendations and upon consent obtained from the Local Ethics Commission of the UWM in Olsztyn, Poland (No. 20/2013/N). Fish were injected intraperitoneally with the colchicine solution and kept in well-aerated aquaria for 1.5 h; they were then sacrificed using an overdose of anesthetics (MS-222 dose 0.15 g L^−1^). Kidneys were taken from the dead fish.

In total, 20 individuals of the common nase were studied: 15 juveniles, 2 females and 3 males. Among them, 17 individuals (12 juveniles, 2 females and 3 males) were captured using nets from the Wisłok River (49°51′57″ N, 21°47′08″ E), the Vistula River drainage area (Baltic Sea basin) by electrofishing, before being transported alive to the laboratory in the Department of Zoology. Furthermore, three juveniles were the result of induced reproduction between *Chondrostoma nasus* females and males collected from the Martwa Wisła (54°24′36″ N, 18°39′39″ E), the Vistula River drainage area (Baltic Sea basin). These rivers are separated from each other by c. 620 km and by several hundred kilometers through their connection with the Vistula River. As is typical for *Chondrostoma nasus*, the specimens examined had a straight mouth, a lower lip with a thick cornified sheath, a dorsal fin with 9.5 branched rays, an anal fin with 10–11.5 branched rays, and red pectoral, pelvic, anal and caudal fins [27,29].

Mitotic chromosome preparations were made from each individual as described by Boroń et al. [33]. First, live fish were injected with a dose of 1 mL of 0.05% colchicine solution per 100 g of body weight. Mitotic chromosomes were obtained from kidney cell suspensions using the air-drying method. The kidney cells were exposed to a hypotonic solution (0.075 M KCl) for 30 min and fixed in methanol:acetic acid (3:1). Chromosomes were stained with a solution of 4% Giemsa (pH = 6.8) and were counted in at least 25 metaphase plates from each fish. Chromosome morphology was determined according to Levan et al. [34], and thus, they were classified as metacentric (m), submetacentric (sm), subtelocentric (st), and acrocentric (a), and were arranged according to karyotype in a decreasing size order.

The silver nitrate (AgNO_3_) staining technique was used to detect the nucleolus organizer regions (NORs) which were functionally active in the preceding interphase. The NORs represent the chromosomal regions where the multiple copy clusters of major (18S, 5.8S, and 28S) ribosomal genes (rDNAs) are located. Chromomycin A_3_ (CMA_3_) staining detects chromosomal regions that contain GC-rich DNA [35]. Chromosome slides of two females, three juveniles and three males were sequentially stained with AgNO_3_ and CMA_3_, according to Sola et al. [35]. C-banding was carried out according to Sumner [36]. At least 15 metaphase plates from each individual were analyzed using MultiScan software (Computer Scanning Systems) with the Karyotype supplement.

The fluorescence in situ hybridization (FISH) procedure described by Boroń et al. was used [33]. The loach 28S rDNA probe was labeled by digoxigenin-11-dUTP (Roche) while the 5S rDNA probe was amplified by PCR, as described by Kirtiklis et al. [37], and labeled by nick translation with biotin-16-dUTP (20-deoxyuridine 5′-triphosphate, Roche). The procedure was performed with RNase-pretreated and formamide-denatured chromosome slides, followed by hybridization with 200 ng of rDNA probes per slide. Post-hybridization and washing in 70% low stringency conditions (37 °C, 20 min), chromosome slides were subjected to detection with anti-digoxigenin-rhodamine (Roche) and avidin-fluorescein isothiocyanate (FITC; Roche, Basel, Switzerland) for 28S and 5S rDNA probes, respectively. Slides were counterstained with 4′, 6-diamidino-2-phenylindole dihydrochloride (DAPI; Vector). Hybridization signals in at least 15 metaphase plates for each individual were observed under a Nikon Eclipse 90i fluorescence microscope using the set of filters for DAPI, FITC, and rhodamine. Dual-color FISH images were taken using a high-resolution ProgRes MFcool camera (Jenoptik) and processed using Lucia software 2.0 (Laboratory Imaging). Voucher specimens were preserved, frozen, and deposited at the Department of Zoology, University of Warmia and Mazury in Olsztyn, Poland.

## 3. Results

### 3.1. Karyotype and Banding Patterns

**Figure 1:**
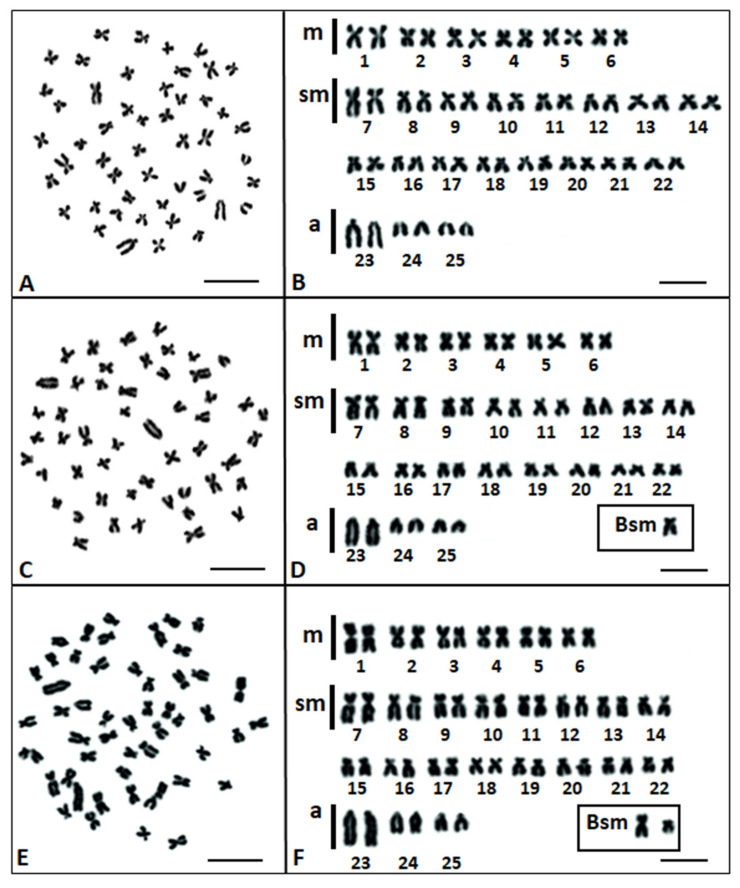
Metaphase plates and karyotypes of the common nase (*Chondrostoma nasus*) with 2*n* = 50 chromosomes (**A**,**B**); 2*n* = 51 chromosomes (**C**,**D**); and 2*n* = 52 chromosomes (**E**,**F**); chromosomes are classified as metacentric (m); submetacentric (sm); acrocentric (a); B chromosomes (Bsm) are shown in Boxes. (bar = 10 µm).

In total 796 Giemsa stained metaphase plates of 20 individuals were counted and analyzed. The majority (83.3%) contained 50 chromosomes, whereas a further 10.7% and 6.0% contained 51 and 52 chromosomes, respectively (Table 1). The individuals of the common nase (*Chondrostoma nasus*) showed a standard (A) chromosome set consisting of 2*n* = 50 chromosomes: 12 metacentric, 32 submetacentric, and 6 acrocentric (Figure 1A,B). The chromosome arm number (NF) was 94. The first metacentric pair (pair No. 1), the first submetacentric pair (pair No. 7), and acrocentric (pair No. 23) pairs were the biggest in the karyotype and similar in size. Acrocentric pairs (pair No. 23) were easily morphologically recognizable among the chromosomes (Figure 1A–F). Six individuals, five juveniles and one female, possessed a karyotype consisting of an A chromosome set (Table 1).

Contrarily, 14 individuals (70%) out of the 20 analyzed showed 1–2 mitotically unstable submetacentric B chromosomes of different sizes (Table 1; Figure 1C–F). Six of these (three juveniles from the Wisłok river and three from the Martwa Wisła River) had a single, medium-sized submetacentric B chromosome (Bsm) in 14.1% of the 248 analyzed metaphase plates (Table 1; Figure 1D). Bs were present in 7.4–19.4% of metaphases from each individual. In turn, four juveniles and all three males possessed a Bsm (2*n* = 51) and a second small B submetacentric chromosome (2*n* = 52) in 19.4% and 15.5% of the 258 analyzed metaphase plates, respectively (Table 1; Figure 1E,F). One and two Bs were present in 5.9–29.5% and 5.7–34.1% of metaphases from each individual, respectively. The small Bsm chromosome was the smallest in the chromosome set 2*n* = 52 of *Chondrostoma nasus* (Figure 1E,F). The remaining *Chondrostoma nasus* female was characterized by both Bsm chromosomes (2*n* = 52) in 14.3% of 56 metaphase plates (Table 1). None of the 20 karyologically analyzed *Chondrostoma nasus* individuals showed heteromorphic sex chromosomes.

**Figure 2:**
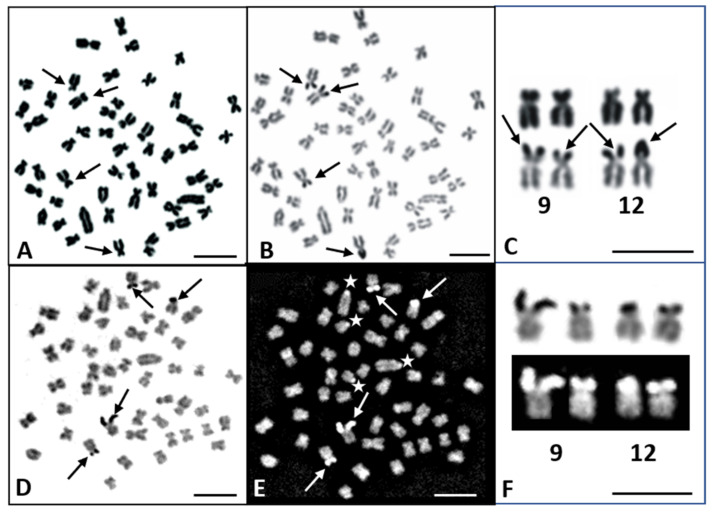
Metaphase plates of the common nase (*Chondrostoma nasus*) with 2*n* = 52 chromosomes (**A**,**B**) and 2*n* = 50 chromosomes (**D**,**E**) stained with Giemsa (**A**) and sequentially using AgNO_3_ (**B**); stained with AgNO_3_ (**D**) and sequentially with CMA_3_ (**E**); silver nucleolar organizer regions (Ag-NORs) (**C**) and CMA_3_-positive (**F**) sites on the chromosome pairs No. 9 and 12. Arrows indicate: Ag-NOR carrying chromosomes (**A**); Ag-NOR sites (**B**,**C**,**D**) and corresponded CMA_3_-positive sites (**E**). Stars indicate other CMA_3_ positive sites (**E**). (Bar = 10 µm). Arrows indicate: Ag-NOR carrying chromosomes (**A**); Ag-NOR sites (**B**,**C**,**D**) and corresponded CMA_3_-positive sites (**E**). Stars indicate other CMA_3_ positive sites (**E**).

The Ag-NORs were in the telomeric position on the short arms of two submetacentric chromosome pairs (No. 9 and 12; Figure 2A–D). However, only 10% of metaphases displayed four signals. Ag-NORs demonstrated intrapopulation variation; from 1 to 4 such positive NOR signals were detected, with pair No. 9 showing the principal NOR activity, and pair No. 12 sometimes showing one Ag-NOR-positive signal. No Ag-NORs were observed in the B chromosomes. In more than 70% of metaphases, four GC-rich DNA sites corresponding with AgNORs were detected by CMA_3_ (Figure 2E,F). Other metaphases displayed three (24%) or less CMA_3_-positive sites. Apart from the CMA_3_-positive signals in the NOR sites, at least four other such signals in the centromeric region of the largest (pair No. 23) and smallest acrocentric chromosome pairs were observed (Figure 2E).

**Figure 3:**
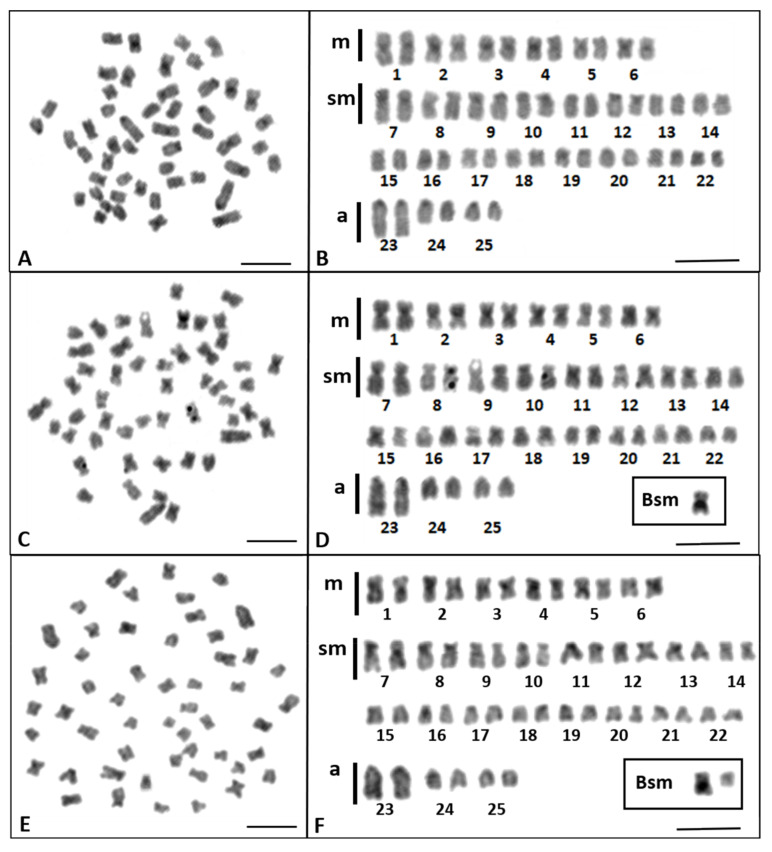
Metaphase plates and karyotypes of the common nase (*Chondrostoma nasus*) with 2*n* = 50 chromosomes (**A**,**B**); 2*n* = 51 chromosomes (**C**,**D**); and 2*n* = 52 chromosomes (**E**,**F**) with C-banding; submetacentric B chromosomes (Bsm) are shown in Boxes. (Bar = 10 µm).

The distribution of constitutive heterochromatin was visible as C bands in the centromeric regions of almost all *Chondrostoma nasus* chromosomes (Figure 3A–F) and in the pericentromeric region of some chromosome pairs (e.g., No. 1–4 and 11) and the larger Bsm chromosome, present in the karyotype of 2*n* = 51 (Figure 3D) and 2*n* = 52 chromosomes (Figure 3F).

### 3.2. FISH Mapping of 28S and 5S rDNA Loci

**Figure 4:**
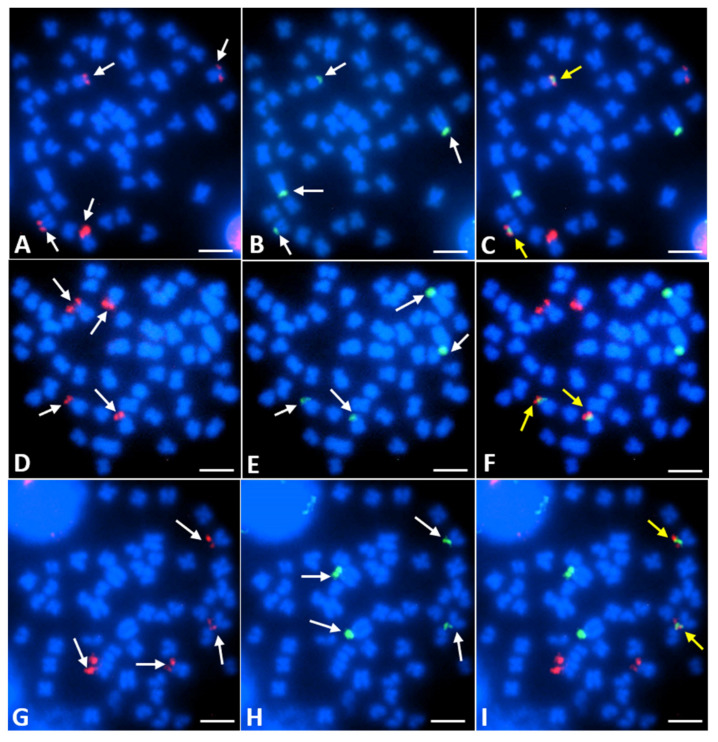
Metaphase plates of the common nase (*Chondrostoma nasus*) with 2n = 50 chromosomes (**A**–**C**); 2*n* = 51 chromosomes (**D**–**F**); and 2*n* = 52 chromosomes (**G**–**I**) after fluorescence in situ hybridization (FISH) analysis with 28S rDNA as a probe (**A**,**D**,**G**); 5S rDNA (**B**,**E**,**H**); and both of these sequences (**C**,**F**,**I**). 28S rDNA (red signals) and 5S rDNA (green signals) hybridization sites are shown by white arrows, whereas co-localizations of both these sequences are indicated by yellow arrows. (Bar = 10 µm).

The 28S and 5S rDNA were analyzed in a subsample of specimens as reported in Table 2. In most of the metaphase plates (85%), the 28S rDNA hybridization signals were found in the short arms of four sm chromosomes, regardless of the number of chromosomes (50, 51, or 52) (Table 2; Figure 4). Therefore, consistent with Ag-NOR patterns, submetacentric chromosome pairs No. 9 and 12 carried a 28S rDNA cluster in the short arm. In most of metaphase plates, one of the 28S rDNA hybridization sites was commonly observed as an intense and broad signal, corresponding with Ag-NOR and CMA_3_ patterns, whereas the signals in the three other sites were less intense (Figure 4A,D,G).

The analysis using FISH with a 5S rDNA probe, revealed two to four loci (Table 2). All individuals showed four such loci (Figure 4B,E,H) in 92.8% of metaphase plates. They were located on the short arms of one submetacentric chromosome pair (pair No. 12, as shown in Figure 1C,F) and in a subcentromericsms position of one acrocentric chromosome pair (pair No. 23), which was the biggest pair of the acrocentric chromosomes (Figure 1). The obtained hybridization signals clearly differed in size and could be classified as larger for the two acrocentric chromosomes and smaller for the submetacentric chromosomes (Figure 4B,E,H). Two and three 5S rDNA hybridization sites were counted in 3.6% of metaphase plates (Table 2). Both classes of rDNA probes were often (in 86.7% of metaphase plates) co-localized on the short arms of one submetacentric chromosome pair (Figure 4C,F,I), which was identified as pair No. 12 in the *Chondrostoma nasus* karyotype.

Thus, *Chondrostoma nasus* is characterized by four 28S and 5S rDNA loci, and two in the synteny. Signal heteromorphism in the 28S rDNA sites, which was reflected in the heteromorphism of the active AgNORs, was detected on chromosome pair No. 9 (Figure 1F and Figure 4A,D,G). No sex-dependent variability in the cytogenetic features was found.

## 4. Discussion

The *Chondrostoma nasus* karyotype (2*n* = 50) presented in this study differs from that given by Barshiene [28] by the greater number of meta- and submetacentric chromosomes and the smaller number of acrocentric chromosomes. The species of the family Leuciscidae, of which *Chondrostoma nasus* is an example [13]*,* are characterized by a stable diploid number (2*n* = 50) and conservative karyotype patterns in most of the analyzed species [32]. These chromosomes can be easily arranged into morphological categories in comparison with the representatives of the family Cyprinidae [38]. Species belonging to *Chondrostoma* s. l., from the genus *Chondrostoma, Achondrostoma*, *Iberochondrostoma*, *Parachondrostoma*, *Protochondrostoma,* and *Pseudochondrostoma,* possess a diploid chromosome number of 2*n* = 50, including 15 to 23 meta- and submetacentric chromosome pairs and 2 to 10 subtelo-acrocentric chromosome pairs [32]. *Chondrostoma regium* is an exception with a diploid number of 2*n* = 52 [39].

The demonstrated presence of B chromosomes in *Chondrostoma nasus* from the Vistula River basin confirms the previous data of individuals (2*n* = 51) from the Zeljeznica river in Bosnia-Herzegovina [26] and indicates that this species joined almost 100 other fish taxa characterized by Bs chromosomes. The category of the Bs may be different among species of one genus, e.g., in the genus *Astyanax*, a large metacentric chromosome was observed [40,41], whereas in several *A*. *scabripinnis* populations, large metacentric, submetacentric and a small metacentric chromosomes were found [42]. In turn, the Bs of *A. alburnus* show either a metacentric or submetacentric morphology [14,17]. Interestingly, the size of the larger *Chondrostoma nasus* B chromosome is similar to a medium-sized submetacentric chromosomes, whereas the second is clearly smaller than the A chromosomes. Regarding two other leuciscid species, the B chromosomes of *R. rutilus* are similar in size to the A set [14,17], whereas *A. alburnus* possesses extremely large supernumerary chromosomes that they are larger than the largest elements of the A set [14,17]. Thus, *Chondrostoma nasus* has joined the fish group possessing large B chromosomes.

In some species B chromosomes were found to be more frequent in females than in males or limited to males [4,43], whereas the Bs of *Chondrostoma nasus* were present in one female and males as well as in most juveniles. However, as the analyzed females differed in the presence of Bs, the research could be repeated on sexually mature individuals.

B chromosomes were found to be mostly heterochromatic [4,43] and, for example, *A. alburnus* characterized by one or two heterochromatic Bs, consisting of GC-rich DNA [14,17]. In turn, *M. sanctaefilomenae* is characterized by two B chromosome variants differing in their C-banding patterns, frequency, and abundance of 18S rDNA [3]. The common nase is different because chromosome B does not contain rDNA sequences but characterized by a large C-band in its pericentromeric region. The 28S rDNA hybridization sites in the karyotype of this species correspond with the Ag-NORs and CMA_3_-positive sites, and regardless of the chromosome number (2*n* = 50, 2*n* = 51, 2*n* = 52), they were located in two submetacentric chromosome pairs, but not in B chromosomes.

Although the number of active Ag-NORs in the karyotype of *Chondrostoma nasus* varied from one to four, FISH with a 28S rDNA probe detected all NOR clusters, both active and non-active. Two pairs of NOR-bearing chromosomes in the karyotype of the common nase represent a derived condition, i.e., an apomorphic state for Teleostei, whereas one pair of subtelocentric chromosomes with NOR sites on the short arms represent a plesiomorphic state. Karyotypes of Leuciscidae species are usually characterized by a single small submetacentric chromosome pair with NORs on the short arms [32,44]. However, some of *Leuciscus* species characterize by multiple NOR patterns [33,37] and the chondrostomine species possess two to four chromosome pairs bearing NORs [32,45]. The second pair of NOR-carrying chromosomes may be the translocation result of rDNA region from one ancestral NOR-bearing chromosome pair to another pair, as was suggested in the evolution of the *C. lusitanicum* karyotype [31]. This relative species, currently named *Iberochondrostoma lusitanicum* (Collares-Pereira, 1980), shares several cytogenetic features with *Chondrostoma nasus,* such as karyotype pattern, the number and location of Ag-NOR regions, and the size polymorphism of Ag-NOR and CMA_3_-positive sites on the submetacentric chromosome pair [32,46]. However, the compared species differ in the number of 28S rDNA hybridization sites that in the karyotype *I. lusitanicum*, there are usually three or four of such sites [30,31]. Similarly, the karyotypes of other species of *Chondrostoma* s. l. are mainly characterized by two, or from two to four, CMA_3_-positive sites, and by two AgNO_3_ as well as three or four 45S rDNA sites [32].

Concerning the number of 5S rDNA hybridization sites, the karyotype of *Chondrostoma nasus* is similar to certain related *Chondrostoma* s. l. species, but differently, they have four such sites terminally located on the subtelo-acrocentric chromosomes, and this pattern is suggested as the plesiomorphic state in Iberian chondrostominae species [32]. It is worth emphasizing that in all these species, the largest acrocentric chromosome pair (pair N. 23 in the karyotype of *Chondrostoma nasus*) contained 5S rDNA sites. Interestingly, to the best of our knowledge, 5S and 28S rDNA sites in a syntenic position on one chromosome pair is documented for the first time.

Although the 5S and 28S rDNAs were not found in the B chromosome of *Chondrostoma nasus,* their small size may have hindered detection by FISH; such is case with *C. gomesi*, which contains 5S rDNA copies but in arrays shorter than the minimum detectable size with this technique [23]. Moreover, in most cases, the DNA composition of B chromosomes is unknown, though in general, they are rich in several classes of repetitive DNA, including 5S and 45S ribosomal DNA, satellite DNA, histone genes, small nuclear DNA, mobile elements, and organellar sequences [1,23,25]. Presumably, the Bs of *Chondrostoma nasus,* such as in other animals and plants, gain their transmission advantage by exceeding the regular rate during cell division via the drive mechanism, which is not well understood [5,47]. Thus, B chromosomes are very promising structures that they can persist inside the cell and are an additional genomic compartment for evolution [23,47]. Therefore, further studies of *Chondrostoma nasus* individuals from other populations, the behavior of B chromosomes in diplotene cells, and the molecular structure and origin of their DNA sequences in relation to those of the A set are required.

## 5. Conclusions

The results obtained in this study significantly improve our knowledge in the field of B chromosome distribution and provide molecular cytogenetics of *Chondrostoma nasus*: a freshwater species from the Leuciscidae family. Most of the analyzed individuals of this species from two populations in the Vistula River drainage area, the Baltic Sea basin showed 1–2 mitotically unstable submetacentric B chromosomes. The detected in the A set, two pairs of NOR-carrying chromosomes and two pairs with 5S rDNA sites seem to be characteristic of *Chondrostoma* related species. In contrast, the 5S and 28S rDNA hybridization sites in a syntenic position on one chromosomes pair are a species-specific feature. The results herein are an example of the benefits of conducting chromosomal investigations, which act to stimulate further studies using more advanced molecular biology techniques.

## Figures and Tables

**Table 1:** The frequency of B chromosomes in the karyotype of *Chondrostoma nasus*; Number of analyzed metaphase plates = *n*.

Number of Fish and Sex	*n*	Number of Metaphase Plates with the Indicated Number of B Chromosomes		
		0 (2*n* = 50)	1 (2*n* = 51)	2 (2*n* = 52)
Five juveniles	193	193	-	-
Female	41	41	-	-
Three juveniles	174	152	22	-
* Three juveniles	74	61	13	-
Four juveniles	170	108	33	29
Three males	88	60	17	11
Female	56	48	-	8

* Individuals from the Martwa Wisła River.

**Table 2:** The frequency of 28S and 5S rDNA hybridization sites in the karyotype of *Chondrostoma nasus*. Number of analyzed metaphase plates = *n*.

Number of Fish and Sex	Number of Metaphase Plates with the Indicated Number of								Co-Localization of 28S and 5S rDNA Sites	
		28S rDNA Sites				5S rDNA Sites				
	*n*	2	3	4	n	2	3	4	1	2
Three juveniles	45	4	-	41	45	2	-	43	4	41
Two females	30	4	2	24	39	3	2	34	8	22
Three males	45	5	3	37	56	-	3	53	4	41
	120	13	5	102	140	5	5	130	16	104
